# Improvement in Attention Processing After Surgical Treatment in Functional Pituitary Adenomas: Evidence From ERP Study

**DOI:** 10.3389/fneur.2021.656255

**Published:** 2021-10-01

**Authors:** Chenglong Cao, Yujing Huang, Aobo Chen, Guozheng Xu, Jian Song

**Affiliations:** ^1^Department of Cognitive Neuroscience, Faculty of Psychology & Neuroscience, Maastricht University, Maastricht, Netherlands; ^2^The First School of Clinical Medical University, Southern Medical University, Guangzhou, China; ^3^Key Laboratory of Structural Biology of Zhejiang Province, Westlake University, Hangzhou, China; ^4^Department of Neurosurgery, Central Theater Command General Hospital of the Chinese People's Liberation Army, Wuhan, China

**Keywords:** pituitary adenoma, event-related potentials, P200, attention processing, improvement

## Abstract

Cognitive abilities are impaired in patients with pituitary adenoma. However, studies on attention processing impairment in preoperative patients and attention processing recovery after transsphenoidal adenomectomy are lacking. The study aims to identify the electrophysiological change that relates to attention processing in pituitary patients before and after treatment. Twenty five preoperative pituitary patients and 25 follow-up postoperative patients were recruited. 27 healthy controls (HCs) were matched to the patients with age, gender, and education. Event-related potentials were used to investigate the attention processing in the preoperative patients, postoperative patients, and HCs. Across three groups, all emotional stimuli evoked P200 components. Compared with the HCs or postoperative patients, the amplitudes of P200 in the preoperative patients were higher. Moreover, The amplitudes of P200 decreased in the postoperative patients, which were similar to that in the HCs. The attention processing was improved after surgery, but no significant differences were detected between the postoperative patients and HCs. Abnormal hormone levels may be relevant to the factor that impair attention processing. Compared with that of the HCs and postoperative patients, the P200 component elicited by negative stimuli is higher in preoperative patients, which may illustrate compensatory activity after attention impairments. Furthermore, these data indicate that improvements in attention processing may be attributed to the amelioration of endocrine disorders. This study shows that the P200 component may be used to diagnose attention processing in preoperative pituitary patients and prove the improvement of attention processing in postoperative patients.

## Background

Pituitary adenomas are the most common intracranial tumors following meningiomas, accounting for about 16.5% of central nervous system tumors (1), and pituitary adenoma with inconspicuous symptoms have a higher incidence. The mechanical pressure from tumor mass on adjacent neuroanatomical regions such as the inferior frontal lobe, diencephalon, optic chiasma, and pituitary stalk could disrupt tissue structures (2–5), which might decrease the endocrine functions of hypothalamus or pituitary stalk. In previous studies, neuropsychological deficits have been identified in traumatic brain injury (TBI) patients (6, 7). Hormone deficits are thought to be the major cause of diverse post-traumatic physical and psychological complaints (8). But, Neuropsychological problems were detected in 67 percent of TBI patients, and they were linked to intracerebral hemorrhagic lesions rather than post-traumatic pituitary insufficiency (9). On the other hand, the pituitary may secrete abnormally high hormones than usual. Therefore, apart from the physical damages, abnormal hormone levels could impair the cognition functions in pituitary adenoma patients. Pituitary patients frequently complain about dysfunction in attention processing (3, 10), which affects the quality of patients' lives. Our team has found that pre-attentive and executive functions are influenced by pituitary adenomas (11–13). Moreover, many studies on affective neuroscience have identified neural networks distributed in the brain and involved in the processing of emotional expressions on the face. These neural networks include the prefrontal, basal ganglia and amygdala regions, which contribute to emotional detection and semantic processing (14).

There are various neuropsychological and behavioral tests to assess cognitive functions (15–17). Since the relationship between the event-related potentials (ERPs) and cognitive performance was identified, many researchers began to study the clinical significance of ERPs to understand the electrophysiological mechanisms of cognition (17). Until recently, no electrophysiological studies of attention processing have been conducted on pre and postoperative pituitary patients, which is addressed in the present study by recording and analyzing P200 elicited by emotional stimuli. P200, a part of the early positive ERPs component, has a peak latency from 100 to 200 ms. P200 is an indicator of the attention bias occurring automatically (18), and also sensitive to the emotional stimuli (19). This aesthetic study, analyzing the front-center-parietal electrodes, has shown that less beautiful pictures elicit a higher amplitude of P200 than beautiful ones. This study indicates that at the early stage of an aesthetic experience, negative emotional experience is automatically aroused for less beautiful pictures. The observed modulation of P200 amplitudes was interpreted as discrepancies in the early emotion evaluation stage. These previous studies have shown that P200 is produced immediately after the detection of threatening stimuli, such as frightening images (20). Hence, P200 can be applied in the study of attention and emotion processing.

In the current study, we aimed to examine whether the function of attention processing was improved by comparing the performances of pre-operative to post-operative pituitary patients. ERPs were used for recording the brain potentials of emotional stimuli in a passive viewing paradigm in pre-operative and post-operative pituitary patients. We hypothesized that the pre-operative patients would show smaller amplitudes of P200. Accordingly, we further predicted that the “negative bias” effect of P200 amplitudes would be dampened in pre-operative patients in contrast to healthy controls (HCs). More importantly, postoperative patients would show improved performance on emotion processing and perform equally to the healthy controls (HCs).

## Methods

### Subject Selection

Pituitary patients were recruited in the Department of Neurosurgery, Central Theater Command General Hospital of the Chinese People's Liberation Army after a definite diagnosis. Patients were included if (1) Prolactinoma patients were diagnosed with a prolactin-secreting pituitary tumor (21, 22). GH adenoma patients were characterized by the overproduction of growth hormone (GH) from a hypersecreting pituitary tumor (23). (2) They had undergone surgery by a transsphenoidal approach and, after at least 6 months post-operatively (the shortest postoperative time is 6 months and the longest postoperative time is 12 months), serum PRL were within the normal range (PRL: 2.64–13.13 ng/ml for males and 3.34–26.72 ng/ml for females). (3) Another head MRI, at least 6 months after the operation, was required to ensure that the tumor is completely removed and there is no recurrence. (4) They had no history of craniotomy or radiation therapy. (5) They could complete ERPs tests. Patients were excluded if they (1) had a history of neurologic or psychiatric disorders, (2) had comorbidities that could affect cognitive function, including severe liver, heart, or kidney dysfunction, (3) had severe complications, such as coma, infection, epilepsy, hydrocephalus, and leaking of cerebrospinal fluid, (4) had drug or alcohol abuse [subjects who drink alcohol over 2.0 standard drinks (10 g of pure alcohol) during the day and meet any 2 of the 11 criteria under the DSM-V in the past year] (24), or were on any medications (including oral contraceptives). All patients and HCs had sufficient visual acuity and hearing ability for the study. Considering circadian changes in hormone levels, vein blood samples were collected in the morning between 8:00 and 9:30. Because we only recruited the patients with abnormally high PRL and growth hormone (GH), this research mainly focused on serum PRL (ng/ml) and GH (ng/ml). Tumor size may have underlying effects on our results because studies have shown that the brain structure changes in pituitary patients, which were caused by macroadenomas (25–27). However, the study population was strictly selected to rule out a big tumor size that compresses optic nerves or surrounding brain structures. There were 25 preoperative patients (including 20 patients with prolactin and 5 patients with GH adenoma) and 25 follow-up (minimum for 6 months and maximum for 12 months) postoperative patients. 27 healthy participants were selected into the HCs. The HCs did not have any mental disorders and a family history of mental disease as well as psychoactive substance abuse. There was no statistical difference in age, gender, educational level among the 25 preoperative and 27 HCs (*p* > 0.05) (Table 1). The study was approved by the ethics committee of the Central Theater Command General Hospital of the Chinese People's Liberation Army and informed consents were understood and signed by the patients themselves.

**Table 1:** Demographic and clinical characteristics: preoperative, postoperative patients and healthy controls.

	**Preoperative patients** **(*n* = 25)**	**Postoperative patients** **(*n* = 25)**	**Healthy control** **(*n* = 27)**	***p*** **-value**
Age (years)	35 (30–40)	35 (30–40)	33 (25–38)	0.06^a^
**Gender**				
Male	9	9	8	χ2 = 0.239
Female	16	16	19	0.625^b^
Education (years)	12 (9–16)	12 (9–16)	13 (9–16)	0.327^a^
Serum PRL (ng/ml)	148.24 (56.06–270.00)	10.73 (4.24–19.42)	NA	
Serum GH (ng/ml)	39.09 (27.33–51.00)	0.71 (0.07–2.35)	NA	
Serum E2 (pg/ml)	35.15 (6.93–79.61)	25.43 (5.70–65.67)	NA	
Serum progesterone (ng/ml)	1.17(0.13–2.41)	1.11 (0.24–3.59)	NA	
Serum FSH (mIU/ml)	5.99(2.61–21.30)	6.96 (2.64–20.31)	NA	
Serum LH (mIU/ml)	4.55(1.89–13.36)	2.13 (1.85–9.14)	NA	
Serum testosterone (ng/ml)	0.97(0.12–4.47)	1.58 (0.09-7.27)	NA	
Serum TSH (mIU/ml)	2.11 (0.37–3.91)	2.08 (0.73–3.80)	NA	
Serum cortisol (nmol/l)	300.5 (115.4–446.90)	286.74 (172.50–394.50)	NA	

*Data are presented as mean values and ranges (minimum and maximum values)*.

a *one-way analysis of variance (ANOVA),*

b *Chi-Square Tests*.

*(PRL, prolactin; GH, growth hormone; E2, estradiol; FSH, follicle-stimulating hormone; LH, luteinizing hormone; TSH, thyroid-stimulating hormone. The range of PRL values are for 20 PRL pituitary patients; the range of GH values are for 5 GH pituitary patients; the range of other hormones are for 25 pituitary patients)*.

### Stimuli and Procedure

Affective stimuli were presented in the gray background in the central monitor (the light degree is set to 60 cd/m2). Participants watched the screen 100 cm away from their eyes in a semi-dark room, with a visual angle of 4° × 4°. Forty-five pictures (15 negative, 15 neutral, and 15 positive pictures) were selected from IAPS (International Affective Picture System) images (28). Table 2 represents the means and standard deviations of both dimensions (arousal and valence) for each type of stimulus.

**Table 2:** Means and standard deviations for arousal and valence for each IAPS.

**Mean valence and arousal ratings**	**IAPS**			
	**Positive**	**Neutral**	**Negative**	***p*** **-value**
Valence	6.1540 ± 1.7842	4.9400 ± 0.1812	3.188 ± 0.6594	< 0.01
Arousal	5.4160 ± 0.3135^a^	2.5673 ± 0.5062	5.4887 ± 0.3905^a^	< 0.01

a *P < 0.05, compared with neutral stimuli*.

One-way analysis of variance (ANOVA) was computed for valence and arousal dimensions. The positive and negative pictures were matched for perceived arousal. They did not differ significantly from each other but differed significantly from the neutral ones (*P* > 0.05). As for valence, positive pictures were significantly higher than neutral ones (*P* < 0.01) and neutral pictures were significantly higher than negative ones (*P* < 0.01). On each trial, there was only one image (positive, negative, or neutral image) presented on the center of the screen.

**Figure 1:**
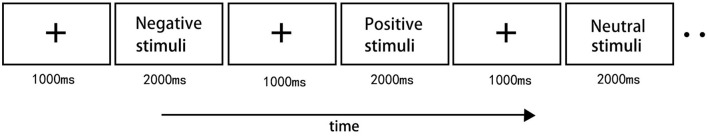
Illustration of the stimulus paradigm applied. There were total 45 images for three stimulus type. 15 positive images, 15 neutral images and 15 negative images were presented with pseudorandom order. The trial began with a fixation mark on the black screen for 1,000 ms, and then an image appeared for 2,000 ms. There were 3 blocks in total.

The trial began with a fixation mark (+) on the black screen for 1,000 ms, and then an image appeared for 2,000 ms (Figure 1). Ten pictures, different from the experimental trials, served as practice trials. Participants were instructed to view each picture attentively and mentally categorize it either as a positive, negative or neutral picture after stimulus onset. Pictures were presented in a randomized order in three blocks, with 45 pictures per block, with no more than two pictures of the same stimulus condition being shown in succession. We instructed participants to mentally categorize these stimuli without pressing any buttons and to only pay attention to these stimuli. During the trial, the subjects were instructed to look passively at the pictures, and reduce eye blinks and body movements as much as they can.

### Electroencephalography (EEG) Recording and Analysis

The EEG was acquired by a 64-channel array (eegoTM amplifier, Germany) linked to both earlobe reference electrodes built-in an elastic cap. The impedance levels of EEG recording were under 5 KΩ. EEG signals were continuously recorded with a bandpass of 0.05–200 Hz. The sampling rate was at 1,000 HZ during acquisition, then was resampled at 250HZ. Based on previous reports (29, 30), we performed ANOVAs with anterior-posterior (AP) distribution (three levels: anterior, central, posterior sites) and lateral distribution (three levels: left, midline, right sites) as within-subject factors. The EEG data were offline referenced to an average reference of all electrodes. The EEG segment was between 100 ms pre-stimulus (31, 32) and 1,000 ms post-stimulus, and the baseline was corrected to the mean amplitude of 100 ms pre-stimulus. The negative, neutral and positive epochs were averaged, respectively. At least 40 trials were made for each subject. The filter frequency was 1HZ high-pass, 40HZ low-pass. We used repeated-measures analysis of variance (ANOVA) to acquire effects of stimulus type and analyze whether these effects are different over the electrode sites of the mean amplitudes of P200 and among different groups. The degrees of freedom in our study were corrected with Greenhouse–Geisserε.

## Results

**Figure 2:**
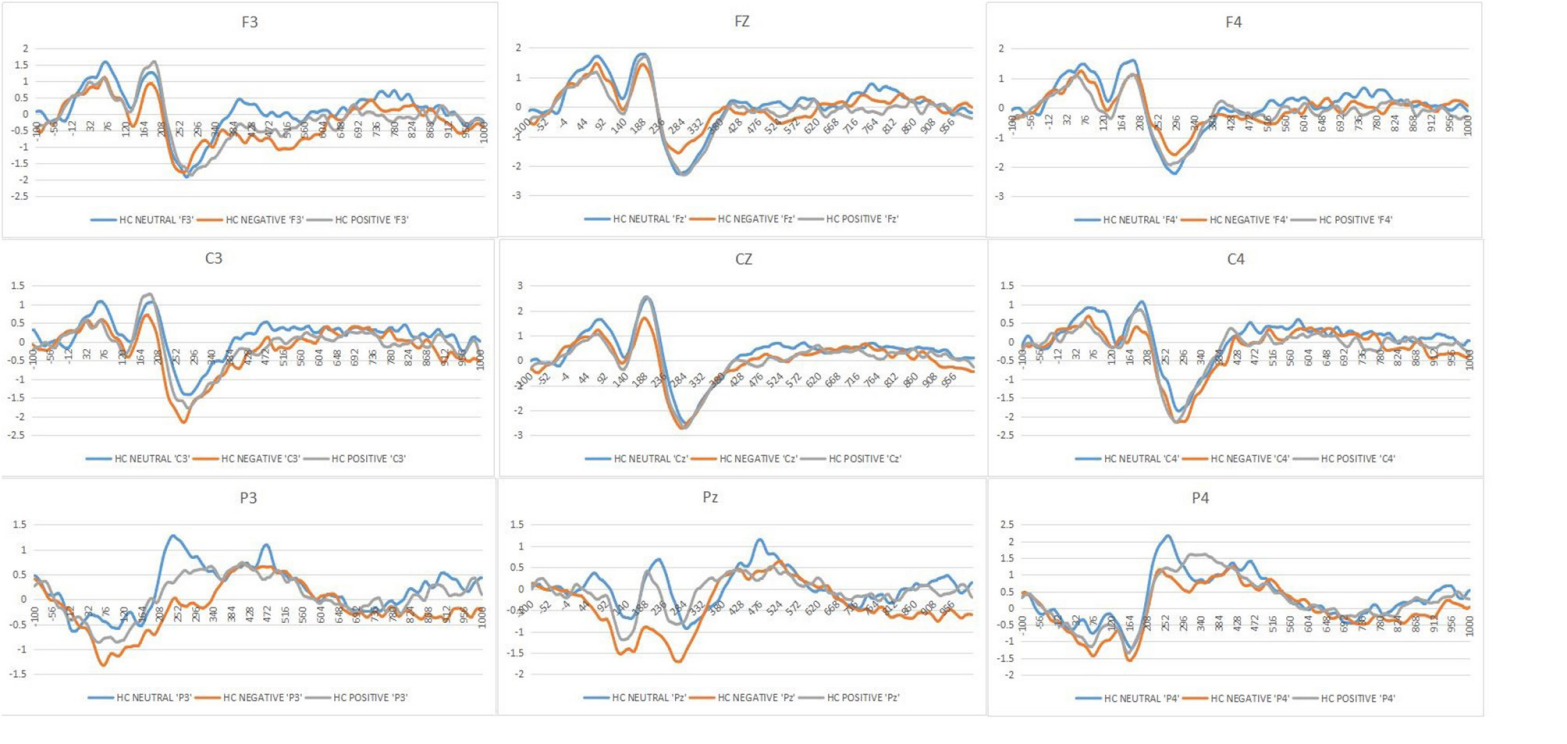
Grand mean ERPs in each affective stimuli and neutral stimuli at the nine ERPs electrodes with time window of P200 for healthy controls (HCs or Healthy controls, Neutral or neutral stimuli, Negative or negative stimuli, Positive or positive stimuli).

**Figure 3:**
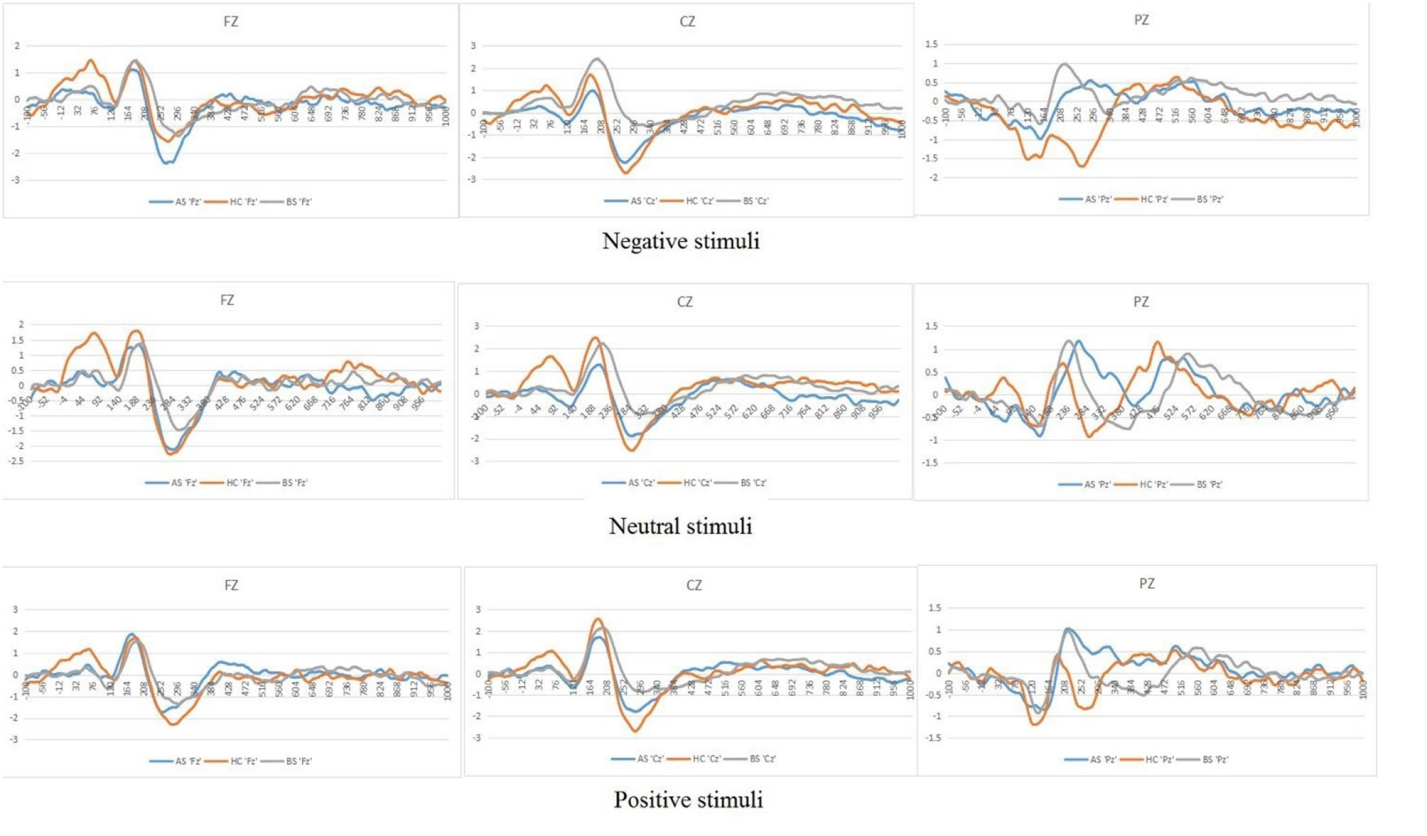
Grand mean ERPs in each emotional stimuli and neutral stimuli at the midline electrodes (Fz, Cz, Pz) with time window of P200 for three groups (AS or postoperative group, BS or preoperative group, HCs or Healthy controls).

**Figure 4:**
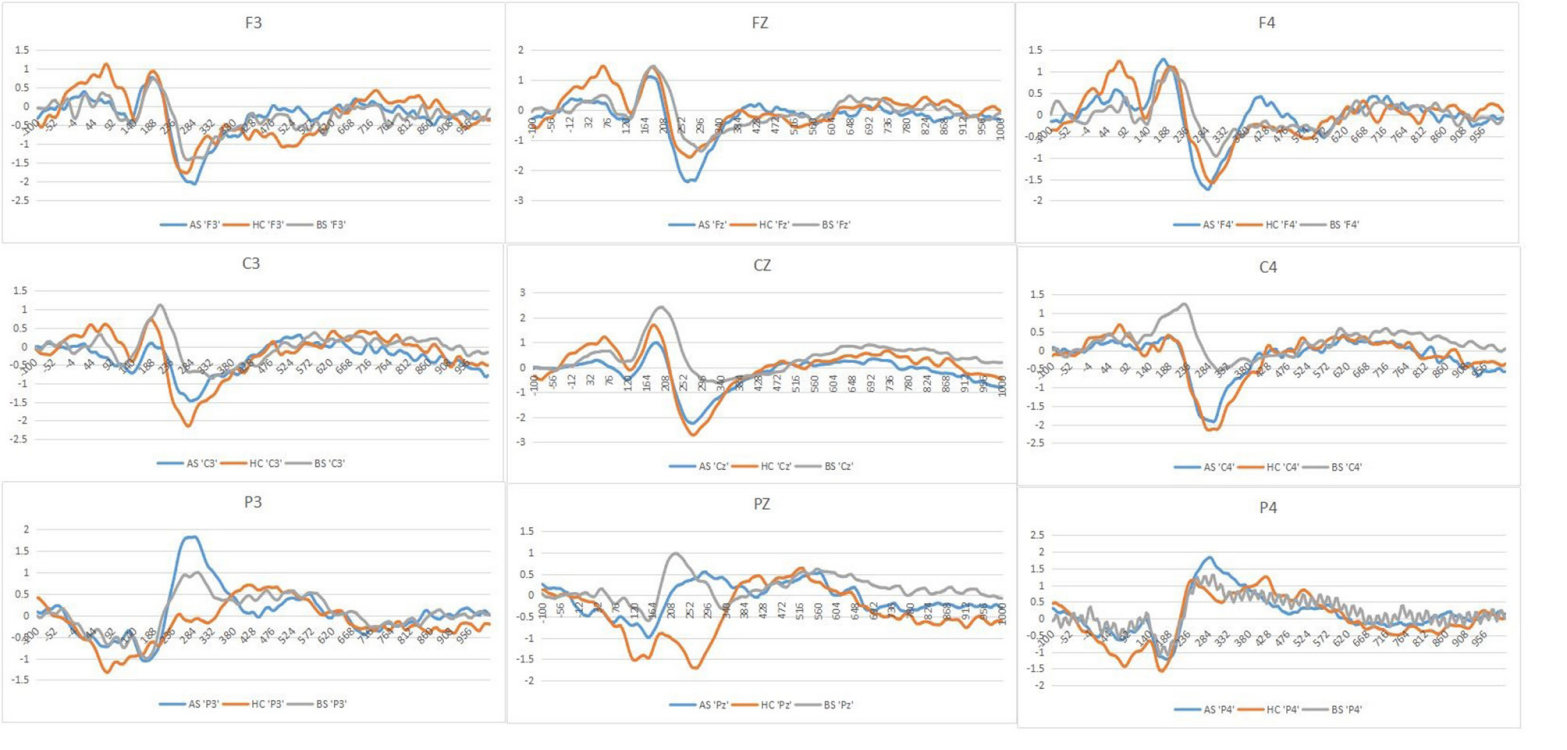
Grand averages in response to negative stimuli of the three groups at each of the nine ERPs electrodes employed in the present experiment stimuli (AS or postoperative group, BS or preoperative group, HCs or Healthy controls).

Figure 2 shows the mean ERPs of HCs at the nine ERPs electrodes within the time window of P200 (148–252 ms) evoked by the three kinds of stimulus, respectively. Compared with the neutral stimuli in the same control group, negative stimuli induced decreased negative potentials in the P200 time window. Figure 3 shows the average ERPs evoked by emotional stimuli and neutral stimuli in the midline areas (Fz, Cz, Pz) in the preoperative group, postoperative group, and the HCs, respectively. Figure 4 shows the mean ERPs of the three groups' responses to negative stimuli at the nine ERPs locations.

### “Negative Bias” of P200 Amplitudes in HCs

With respect to P200 amplitudes, there were no remarkable different effects, though we obtained significant interactions of Stimulus type^*^Group [*F*_(4, 148)_ = 3.14, *p* = 0.01, $\eta_{\text{p}}^{2}$ = 0.07], but no interaction effect of the Hemisphere^*^Group [*F*_(4, 148)_ = 1.68, *p* = 0.17, $\eta_{\text{p}}^{2}$ = 0.04]. *Post-hoc* results showed that the effect of the Stimuli was clearly significant in the HCs [*F*_(2, 73)_ = 7.45, *p* = 0.001, $\eta_{\text{p}}^{2}$ = 0.16]. The negative stimuli (−0.17 μV) evoked decreased amplitudes of P200 compared with the positive and neutral stimuli in the control group (neutral = 0.42 μV, positive = 0.21 μV) (Figure 2).

### Increased Amplitudes of P200 in Preoperative Prolactinomas

We found no interaction effect of Lateral electrodes and Group (*p* = 0.17). However, we observed differences in P200 amplitudes in midline electrodes among pre-operative patients, post-operative patients and HCs. Specifically, *post-hoc* analyses indicated that differences between both preoperative (0.86 μV) and postoperative (0.24 μV, *p* = 0.01) and preoperative (0.86 μV) and HCs (0.24 μV, *p* = 0.02) were significant, but no difference between postoperative (0.24 μV) and HCs (0.24 μV, *p* > 0.05) (Figure 3).

In response to negative stimuli of the three groups, *post-hoc* analyses indicated that the differences between both preoperative (0.53 μV) and postoperative (−0.09 μV, *p* = 0.006) and preoperative (0.53 μV) and HCs (−0.17 μV, *p* = 0.002) were significant, without differences between postoperative (−0.09 μV) and HCs (−0.17 μV, *p* > 0.05) (Figure 4). Furthermore, *post-hoc* analyses revealed decreased amplitudes in the postoperative (0.17 μV), compared to the preoperative (0.91 μV, *p* = 0.04) at central electrode. We did not find any correlation between PRL levels and the amplitude of P200 (*r* = 0.440, *p* = 0.052) and correlation between GH levels and the amplitude of P200 (*r* = 0.488, *p* > 0.05).

## Discussion

In studies of pituitary adenoma to date, much attention has been paid to the effects of surgery. However, few studies have investigated the patient's attention processing and recovery after treatment. Our study found that the HCs manifested decreased P200 amplitude on the negative stimuli. In response to negative stimuli, the amplitude of P200 was more robust in preoperative patients than in the HCs and postoperative patients. Moreover, the amplitude of P200 was larger in the preoperative patients than that in the HCs and postoperative patients at midline regions, which may support a functional compensatory mechanism that occurs before the cascade of structural damage. Here, we also sought to assess the recovery of attention processing in postoperative patients.

The association of the concrete operation and its effects on cognitive functions is still inconsistent (33). For instance, Peace et al. (3) found that the transfrontal operation caused more severe injury to cognition, but Guinan et al. (34) observed no association between the cognitive dysfunction and the treatment approach (3, 34, 35). Therefore, all the patients did not go through the transfrontal operation and radiation treatment but underwent transsphenoidal adenomectomy. In this study, we did not carry out a neuropsychological test. Because our previous research (36) assessed attention processing using the Digit Span Forwards and Backwards Tests of the Wechsler Adult Intelligence Scale-Revised (Chinese version). We did not find any obvious differences between the preoperative group and HCs, perhaps because of the simplicity of the present behavioral task. However, we found significant group differences for electrophysiological data in this research.

This study found that compared with the positive and neutral stimuli, the negative stimuli elicited decreased amplitudes of P200 in the HCs. Previous studies indicated that negative stimuli elicited more prominent responses than neutral or positive stimuli (18, 37). However, recent research has found that the extremely negative (EN) stimuli produced smaller amplitudes of P200 than did the moderately negative (MN) and neutral stimuli over a wide region across the scalp (38). Frontal P200 activation is the sign of quick detection of typical stimuli (39). Relevant to moderately negative stimuli, EN stimuli often include remarkable threatening contents (e.g., bleeding picture existing in our negative stimuli), which have been shown to attract human attention resources rapidly and automatically (40, 41). Moreover, it has been reported that there is a cultural bias for the IAPS in Chinese subjects (42). Pictures containing intense feelings are contradictory with traditional Chinese culture, meaning their valence scores were significantly lower than that of the original subjects (43), which meant these stimuli could be intensely negative for participants. Therefore, smaller P200 amplitudes in the negative condition are likely a rapid feature detection process to threatening content (41). In line with the discussion above, we also found that the negative stimuli elicited decreased amplitudes of P200 in the HCs, which confirmed previous findings.

We obtained the differences among the three groups at midline areas. We found no hemispheric differences in our EEG recordings because the asymmetry is neither very significant in some phases of the emotional reaction nor some stimulus conditions (44, 45). Moreover, in response to negative stimuli of the three groups, this study showed that the amplitudes of P200 in preoperative patients were larger than that in both postoperative patients and HCs. When faced with EN stimuli, such as remarkable threatening stimuli, it is unclear whether preoperative patients have difficulties attracting attention resources rapidly and automatically. Preoperative patients exhibited the larger amplitudes of P200, which may illustrate compensatory activity within the midline cortex related to emotion processing. Our team previously also found that increased thalamocortical and cerebellar-cerebral functional connectivity (FC) was associated with endogenous hormone levels, which supports a functional compensatory mechanism that occurs before the cascade of structural damage (46). Yao et al. found that prolactinoma patients showed increased FC mostly between the posterior brain regions and temporal lobes, namely the cerebellum, precuneus, posterior cingulate cortex (PCC), and bilateral temporal fusiform cortex (TFusC). As a result, enhanced connections of posterior brain regions in these patients might be used as an imaging biomarker for cognitive dysfunctions (47). Although these prolactinomas in the research showed increased FC between these brain regions, there are limitations in dictating whether these patients suffer from the dysfunctions of attention processing due to the absence of tasks that stimulate emotions. Therefore, combined with the results of the present experiment, it could be considered that pituitary patients may have attention processing impairments.

Regarding the characteristics of pituitary patients *per se*, it was reported that pituitary adenoma patients showed a different mode of increased anxious personality compared with the general population (48). Increased P200 amplitudes are reported in high trait-anxious individuals (49). The boost of perceptual processing has also been indicated in other types of phobic syndromes, and such a state of hypervigilance to salient stimuli might be a feature of the cognitive functioning of phobic individuals (50–52). Furthermore, pituitary adenoma patients have a significantly higher degree of depression and anxiety than HCs (52). It may be suggested that the larger amplitudes of P200 could reflect a greater involvement of intense resources on pivotal stimuli in pituitary patients. Because preoperative patients may have difficulties attracting attention resources rapidly and automatically, when they are faced with EN stimuli, such as remarkable threatening stimuli. We postulate that a high degree of depression and anxiety in pituitary adenoma patients decreased processing efficiency through the increased use of neural resources. Therefore, the facilitated responses to negative stimuli in preoperative patients could be these factors. One is the compensatory mechanisms that preoperative patients have involves difficulties attracting attention resources rapidly, meaning they have to employ more neural resources to complete negative stimuli processing. Previous research found that increased P200 amplitudes during attention tasks in depressed participants, which suggested that neural systems were attempting to compensate for the difficulty in perceiving target and non-target stimuli (53). According to our previous findings that prolactinoma patients showed the decreased gray matter volume in the prefrontal cortex, pituitary patients examined early in the disease course (the maximum disease duration <22 months) in the present research might also suffer from brain structure impairments. But the compensatory mechanism described above is beneficial to maintaining normal physiological function, which has been regarded as a common phenomenon in some psychiatric diseases (54–56). Besides, high trait-anxious pituitary patients may apply increased use of neural resources to attend to remarkable threatening stimuli. Hence, the use of the P200 component during presenting affective stimuli could be used as a relevant marker to investigate attention function.

The cognitive dysfunction may be caused by the effect of related hormonal imbalance that influences cognitive structures. However, up to now, the detailed mechanism of cognitive impairments is still unexplained (57, 58). Previous animal experiments have shown that proper PRL level plays a significant role in preventing stress-induced decrease of hippocampal neurogenesis and protects against excitotoxicity to hippocampal neurons through PRL receptors (59, 60). Nevertheless, if the PRL level is too high, it can produce negative effects on cognitive abilities (57, 61–63). These cognitive dysfunctions, especially attention function, are also related to the abnormal prefrontal cortex. Previous studies have suggested that frontal/parietal cortex and subcortical structures (e.g., thalamus) were related to attention selection and shifting (64, 65). Our study also found that patients with prolactinomas showed a decrease of gray matter volume (GMV) in the whole prefrontal cortex (36) suggesting that abnormal high PRL levels may harm cognitive function. In addition, our team has shown increased FC in prolactinomas and quantified the hormone-FC connections. These findings have proved that endogenous hormones are essential for brain functional compensation in these patients. Bala et al. also speculated that the PRL overproduction might influence the efficiency of cognitive functioning via the dopamine pathway (57), which could be altered in prolactinoma patients because of the anti-correlation between dopamine secreting and prolactin production (66). Therefore, patients with abnormally high PRL may have cognitive impairments due to brain structural damages.

Biologically, PRL overproduction may be relevant in neuronal changes and plasticity in the brain cortex because PRL significantly enhances the number of cells secreting antibodies directed against myelin oligodendrocyte glycoprotein (67). Oligodendrocytes are a type of neuroglia whose main function is providing support and insulation to axons in the central nervous system of some vertebrates (68). Accordingly, hypersecretion of PRL can directly affect the brain structures relevant to attention processing. GH can play neuroprotective effects on the brain by the enhanced expression of IGF-I and activating intracellular signal transduction pathways (69). However, overexpression of IGF-1 caused insulin resistance in the brain and promoted the hyperphosphorylation of tau protein and the accumulation of amyloidosis, which will eventually lead to synaptic apoptosis. Patients with high levels of insulin-like growth factor (IGF-I) and GH also show specific cognitive disorders (70), in that GH and IGF-I can cross the brain-blood barrier (71). Gray and white matter volume in the hippocampus of acromegaly patients increase (72), other researchers suggest that increased brain volume mediated by high-level GH/IGF-I is due to gliogenesis and increased glial activity, not due to neurogenesis (73, 74). Hypersecretion of these hormones can directly affect the brain structures relevant to cognitive functions. In this research, we did not find any obvious correlation between PRL levels and the amplitudes of P200 and correlation between GH levels and amplitudes of P200. Our previous findings established that prolactinoma patients suffered from deficits in response activation and inhibition, but there were also no correlations between PRL levels and ERPs amplitudes among specific brain areas (under review). Limited sample size could result in the phenomenon. Besides, if the PRL and GH are not high enough, it may not reach the threshold to elicit a correlation between hormone levels and the amplitudes of P200.

Preoperative patients manifested deficits of attention processing, showing larger amplitudes of P200. However, with physiologically normal levels of the hormone, the postoperative pituitary patients manifested improvement of attention processing, showing similar amplitudes of P200 as the HCs did. Improvement in cognitive functions after surgery was related to the normal endocrine (75). This suggests improvement in attention processing, which could be due to the normalization of hypothalamic-pituitary axes and secretory rhythms of pituitary hormones after removal of the tumor. Besides, during routine treatment, post-operative patients are maintained on the low dose of a dopamine D2 receptor agonist (e.g., cabergoline or Bromocriptine) to control hormones to normal levels. There have been few reports of reversible cognitive impairment related to the therapy of prolactinomas. Bukowczan et al. found that a 22-year old student with an invasive giant prolactinoma following cabergoline treatment showed reversible cognitive impairment due to inducing dramatic tumor reduction (76). A rodent study found that bromocriptine, a dopamine D2 receptor agonist with significant antioxidant properties, attenuated traumatic brain injury-induced lipid peroxidation and provided behavioral and histological protection (77). Bromocriptine therapy after brain injury may provide neuroprotection by minimizing lipid peroxidation. Therefore, the recovery of attention processing in post-operative patients may also be due to a dopamine D2 receptor agonist treatment. In future, continued studies investigating potential mechanisms for its beneficial effects on functional outcomes after treatment of pituitary adenomas are warranted.

Several limitations should be addressed. First, although there was a significantly increased P200 amplitude in the preoperative group and decreased P200 amplitudes in the postoperative group, the relatively small sample size should be increased in future studies to validate the generalization of this finding. Second, the age of the patient at the time may play a role in hormonal disorders because older adolescents are more vulnerable to suffering cognitive impairments compared to young patients (78).

## Conclusion

In summary, the present study investigated the change of attention processing in pituitary adenoma patients by analyzing the P200 component. Preoperative patients showed larger P200 amplitudes than the HCs and postoperative patients, which may illustrate compensatory activity after the attention impairments. Postoperative pituitary patients manifested similar amplitudes of P200 as the HCs did, which suggests the improvement of attention processing. Successful removal of pituitary adenomas and the restoration to normal hormone levels may be associated with the recovery of attention processing.

## Data Availability Statement

The raw data supporting the conclusions of this article will be made available by the authors, without undue reservation.

## Ethics Statement

The studies involving human participants were reviewed and approved by the Institutional Review Board of Central Theater Command General Hospital of the Chinese People's Liberation Army. The number of the approved ethical statement is [2014] 024-1. The patients/participants provided their written informed consent to participate in this study.

## Author Contributions

GX designed and supervised the study, and edited the manuscript. CC supervised the data collection, data analysis, and drafted the manuscript. JS supervised the study and provided specialist knowledge support. YH provided technical and material support. AC contributed to data collection. All authors contributed to the article and approved the submitted version.

## Funding

This work is supported by the funding from National Natural Science Foundation of China (ID: 81571049 and 81870863) and the Chinese Scholarship Council (202008440671).

## Conflict of Interest

The authors declare that the research was conducted in the absence of any commercial or financial relationships that could be construed as a potential conflict of interest.

## Publisher's Note

All claims expressed in this article are solely those of the authors and do not necessarily represent those of their affiliated organizations, or those of the publisher, the editors and the reviewers. Any product that may be evaluated in this article, or claim that may be made by its manufacturer, is not guaranteed or endorsed by the publisher.

## Abbreviations

HCs: healthy controls
ERPs: event-related potentials
MRI: magnetic resonance imaging
DSM-V: Diagnostic and Statistical Manual of Mental Disorders
IAPS: International Affective Picture System
EEG: Electroencephalography
AP: anterior-posterior
ANOVA: analysis of variance
HPA: hypothalamic-pituitary-adrenal
PRL: prolactin
GH: growth hormone
GMV: gray matter volume
FC: functional connectivity.

## References

[1] Ostrom QT Gittleman H Truitt G Boscia A Kruchko C Barnholtz-Sloan JS. CBTRUS statistical report: primary brain and other central nervous system tumors diagnosed in the United States in 2011-2015. Neuro Oncol. (2018) 20:iv1–iv86. 10.1093/neuonc/noy131 30445539 PMC6129949

[2] Kleinschmidt-DeMasters BK Winston KR Rubinstein D Samuels MH. Ectopic pituitary adenoma of the third ventricle. Case report. J Neurosurg. (1990) 72:139–42. 10.3171/jns.1990.72.1.0139 2294174

[3] Peace KA Orme SM Thompson AR Padayatty S Ellis AW Belchetz PE. Cognitive dysfunction in patients treated for pituitary tumours. J Clin Exp Neuropsychol. (1997) 19:1–6. 10.1080/01688639708403831 9071636

[4] Yamamoto T Sakakibara R Uchiyama T Liu Z Ito T Yamanishi T. Lower urinary tract function in patients with pituitary adenoma compressing hypothalamus. J Neurol Neuro Psychiatry. (2005) 76. 390–4. 10.1136/jnnp.2004.044644 15716534 PMC1739555

[5] Tachibana O Yamaguchi N Yamashima T Yamashita J. Radiation necrosis of the optic chiasm, optic tract, hypothalamus, and upper pons after radiotherapy for pituitary adenoma, detected by gadolinium-enhanced, T1-weighted magnetic resonance imaging: case report. Neurosurgery. (1990) 27:640–3. 10.1227/00006123-199010000-00025 2234373

[6] Kreutzer J Vance ML Lopes MBS Laws ER Jr. Surgical management of GH-secreting pituitary adenomas: an outcome study using modern remission criteria. J Clin Endocrinol Metab. (2001) 86:4072–7. 10.1210/jcem.86.9.7819 11549628

[7] Lippert-Grüner M Lefering R Svestkova O. Functional outcome at 1 vs. 2 years after severe traumatic brain injury. Brain Injury. (2007) 21:1001–5. 10.1080/02699050701468933 17891561

[8] Popovic V Pekic S Pavlovic D Maric N Jasovic-Gasic M Djurovic B. Hypopituitarism as a consequence of traumatic brain injury (TBI) and its possible relation with cognitive disabilities and mental distress. J Endocrinol Invest. (2004) 27:1048–54. 10.1007/BF03345308 15754737

[9] Wachter D Gündling K Oertel MF Stracke H Böker DK. Pituitary insufficiency after traumatic brain injury. J Clin Neurosci. (2009) 16:202–8. 10.1016/j.jocn.2008.01.009 19097906

[10] Noad R Narayanan KR Howlett T Lincoln NB Page RC. Evaluation of the effect of radiotherapy for pituitary tumours on cognitive function and quality of life. Clin Oncol. (2004) 16:233–7. 10.1016/j.clon.2004.01.012 15214645

[11] Cao C Wen W Liu B Ma P Li S Xu G. Theta oscillations in prolactinomas: neurocognitive deficits in executive controls. Neuroimage Clin. (2020) 28:102455. 10.1016/j.nicl.2020.102455 33038668 PMC7554198

[12] Cao C Song J Yao S Yan Y Li S Peng G. The dysfunction of inhibition control in pituitary patients: evidence from the Go/Nogo event-related potential study. Neuroreport. (2017) 28:272–8. 10.1097/WNR.0000000000000757 28225481

[13] Song J Cao C Yang M Yao S Yan Y Peng G. The dysfunction of processing task-irrelevant emotional faces in pituitary patients: an evidence from expression-related visual mismatch negativity. Neuroreport. (2018). 29:328–33. 10.1097/WNR.0000000000000965 29369901

[14] Adolphs R. Neural systems for recognizing emotion. Curr Opin Neurobiol. (2002) 12:169–77. 10.1016/S0959-4388(02)00301-X 12015233

[15] Cockrell JR Folstein MF. Mini-mental state examination (MMSE). Austr J Physiother. (2005) 51:689–92. 10.1016/S0004-9514(05)70034-9 3249771

[16] Lindeboom J Ter Horst R Hooyer C Dinkgreve M Jonker C. Some psychometric properties of the CAMCOG. Psychol Med. (1993) 23:213. 10.1017/S0033291700039003 8475210

[17] Sutton S Braren M Zubin J John ER. Evoked-potential correlates of stimulus uncertainty. Science. (1965) 150:1187–8. 10.1126/science.150.3700.1187 5852977

[18] Huang YX Luo YJ. Temporal course of emotional negativity bias: an ERP study. Neurosci Lett. (2006) 398:91–6. 10.1016/j.neulet.2005.12.074 16446031

[19] Wang X Huang Y Ma Q Li N. Event-related potential P2 correlates of implicit aesthetic experience. Neuroreport. (2012) 23:862–6. 10.1097/WNR.0b013e3283587161 22922601

[20] Correll J Urland GR Ito TA. Event-related potentials and the decision to shoot: the role of threat perception and cognitive control. J Exp Soc Psychol. (2006) 42:0–128. 10.1016/j.jesp.2005.02.006

[21] Casanueva FF Molitch ME Schlechte JA Abs R Bonert V Bronstein MD. Guidelines of the pituitary society for the diagnosis and management of prolactinomas. Clin Endocrinol. (2006) 65:265–73. 10.1111/j.1365-2265.2006.02562.x 16886971

[22] Melmed S Casanueva FF Hoffman AR Kleinberg DL Montori VM Schlechte JA. Diagnosis and treatment of hyperprolactinemia: an endocrine society clinical practice guideline. J Clin Endocrinol Metab. (2011) 96:273–88. 10.1210/jc.2010-1692 21296991

[23] Yao S Chen WL Tavakol S Akter F Catalino MP Guo X. Predictors of postoperative biochemical remission in acromegaly. J Neurooncol. (2021) 151:313–24. 10.1007/s11060-020-03669-4 33394265 PMC10077515

[24] American Psychiatric Association DSM-5 Task Force. Diagnostic and Statistical Manual of Mental Disorders: DSM-5, 5th Edn. American Psychiatric Publishing, Inc. (2013). 10.1176/appi.books.9780890425596

[25] Guo X Yao S Xing B. Letter to the editor. Is 7-Tesla MRI necessary in the assessment of microstructural injury to visual pathways due to pituitary adenomas? J Neurosurg. (2019) 132:675–7. 10.1093/biostatistics/kxy047 31100727

[26] Rutland JW Delman BN Huang KH Verma G Benson NC Villavisanis DF. Primary visual cortical thickness in correlation with visual field defects in patients with pituitary macroadenomas: a structural 7-Tesla retinotopic analysis. J Neurosurg. (2019) 1–11. 10.3171/2019.7.JNS191712. [Epub ahead of print]. 31628280 PMC7205160

[27] Yao S Akter F Zhang RY Li Z. Letter to the Editor. Structural retinotopic analysis at 7-Tesla MRI in pituitary macroadenomas. J Neurosurg. (2020) 1–3. 10.3171/2019.11.JNS193149 [Epub ahead of print]. 32059189

[28] Lang PJ Bradley MM. Emotion and the motivational brain. Biol Psychol. (2010) 84:437–50. 10.1016/j.biopsycho.2009.10.007 19879918 PMC3612949

[29] Delogu F Drenhaus H Crocker MW. On the predictability of event boundaries in discourse: an ERP investigation. Mem Cognit. (2018) 46:315–25. 10.3758/s13421-017-0766-4 29159678 PMC5809541

[30] Carretié L Mercado F Tapia M Hinojosa JA. Emotion, attention, and the ‘negativity bias’, studied through event-related potentials. Int J Psychophysiol. (2001) 41:75–85. 10.1016/S0167-8760(00)00195-1 11239699

[31] Champagne J Mendrek A Germain M Hot P Lavoie ME. Event-related brain potentials to emotional images and gonadal steroid hormone levels in patients with schizophrenia and paired controls. Front Psychol. (2014) 5:543. 10.3389/fpsyg.2014.00543 24966840 PMC4052747

[32] Rossignol M Philippot P Bissot C Rigoulot S Campanella S. Electrophysiological correlates of enhanced perceptual processes and attentional capture by emotional faces in social anxiety. Brain Res. (2012) 1460:0–0. 10.1016/j.brainres.2012.04.034 22592075

[33] Donnet A Schmitt A Dufour H Grisoli F. Neuropsychological follow-up of twenty two adult patients after surgery for craniopharyngioma. Acta Neurochir. (1999) 141:1049–54. 10.1007/s007010050481 10550648

[34] Guinan EM Lowy C Stanhope N Lewis PDR Kopelman MD. Cognitive effects of pituitary tumours and their treatments: two ncase studies and an investigation of 90 patients. J Neurol Neurosurg Psychiatry. (1999) 65:870–6. 10.1136/jnnp.65.6.870 9854963 PMC2170410

[35] Peace K Orme SM Padayatty SJ Godfrey HPD Belchetz PE. Cognitive dysfunction in patients with pituitary tumour who have been treated with transfrontal or transsphenoidal surgery or medication. Clin Endocrinol. (1998) 49:391–6. 10.1046/j.1365-2265.1998.00543.x 9861332

[36] Yao S Song J Gao J Lin P Yang M Zahid KR. Cognitive function and serum hormone levels are associated with gray matter volume decline in female patients with prolactinomas. Front Neurol. (2017) 8:742. 10.3389/fneur.2017.00742 29434564 PMC5797301

[37] Delplanque S Silvert L Hot P Sequeira H. Event-related P3a and P3b in response to unpredictable emotional stimuli. Biol Psychol. (2005) 68:107–20. 10.1016/j.biopsycho.2004.04.006 15450691

[38] Yuan J Zhang Q Chen A Li H Wang Q Zhuang Z. Are we sensitive to valence differences in emotionally negative stimuli? Electrophysiological evidence from an ERP study. Neuropsychologia. (2007) 45:2764–71. 10.1016/j.neuropsychologia.2007.04.018 17548095

[39] Thorpe S Fize D Marlot C. Speed of processing in the human visual system. Nature. (1996) 381:520–2. 10.1038/381520a0 8632824

[40] Hansen CH Hansen RD. Finding the face in the crowd: an anger superiority effect. J Pers Soc Psychol. (1988) 54:917–24. 10.1037/0022-3514.54.6.917 3397866

[41] Li X Li X Luo YJ. Anxiety and attentional bias for threat: an event-related potential study. Neuroreport. (2005) 16:1501–5. 10.1097/01.wnr.0000176522.26971.83 16110279

[42] Yuan JJ Yang JM Meng XX Yu FQ Li H. The valence strength of negative stimuli modulates visual novelty processing: electrophysiological evidence from an event-related potential study. Neuroscience. (2008) 157:524–31. 10.1016/j.neuroscience.2008.09.023 18926886

[43] Huang J Xu D Peterson BS Hu J Cao L Wei N. Affective reactions differ between Chinese and American healthy young adults: a cross-cultural study using the international affective picture system. BMC Psychiatry. (2015) 15:60. 10.1186/s12888-015-0442-9 25885052 PMC4378560

[44] Davidson RJ. Cerebral asymmetry, emotion, affective style. Massac Inst Technol. (1995) 12:361–87.

[45] Spence S Shapiro D Zaidel E. The role of the right hemisphere in the physiological and cognitive components of emotional processing. Psychophysiology. (1996) 33:112–22. 10.1111/j.1469-8986.1996.tb02115.x 8851239

[46] Yao S Lin P Vera M Akter F Zhang RY Zeng A. Hormone levels are related to functional compensation in prolactinomas: a resting-state fMRI study. J Neurol Sci. (2020) 411:116720. 10.1016/j.jns.2020.116720 32044686 PMC7096250

[47] Guo W Liu F Zhang Z Liu G Liu J Yu L. Increased cerebellar functional connectivity with the default-mode network in unaffected siblings of schizophrenia patients at rest. Schizop Bull. (2015) 41:1317–25. 10.1093/schbul/sbv062 25956897 PMC4601712

[48] Sievers C Ising M Pfister H Dimopoulou C Schneider HJ Roemmler J. Personality in patients with pituitary adenomas is characterized by increased anxiety-related traits: comparison of 70 acromegalic patients with patients with non-functioning pituitary adenomas and age- and gender-matched controls. Eur J Endocrinol. (2009) 160:367–73. 10.1530/EJE-08-0896 19073833

[49] Bar-Haim Y Lamy D Glickman S. Attentional bias in anxiety: a behavioral and ERP study. Brain Cogn. (2005) 59:11–22. 10.1016/j.bandc.2005.03.005 15919145

[50] Frauke M Iris-Tatjana K Stephan K Miltner Wolfgang HR. Event-related potentials when identifying or color-naming threatening schematic stimuli in spider phobic and non-phobic individuals. Bmc Psychiatry. (2006) 6:38. 10.1186/1471-244X-6-38 16981991 PMC1618387

[51] Kolassa IT Kolassa S Bergmann S Lauche R Dilger S Miltner WHR. Interpretive bias in social phobia: an ERP study with morphed emotional schematic faces. Cog Emot. (2009) 23:69–95. 10.1080/02699930801940461

[52] Ragnarsson O Berglund P Eder DN Johannsson G. Long-term cognitive impairments and attentional deficits in patients with Cushing's disease and cortisol-producing adrenal adenoma in remission. J Clin Endocrinol Metab. (2012) 97:E1640–8. 10.1210/jc.2012-1945 22761462

[53] Kemp AH Hopkinson PJ Hermens DF Rowe DL Sumich AL Clark CR. Fronto-temporal alterations within the first 200 ms during an attentional task distinguish major depression, non-clinical participants with depressed mood and healthy controls: a potential biomarker? Human Brain Map. (2009) 30:602–14. 10.1002/hbm.20528 18181154 PMC6870851

[54] Chiaravalloti ND Genova HM DeLuca J. Cognitive rehabilitation in multiple sclerosis: the role of plasticity. Front Neurol. (2015) 6:67. 10.3389/fneur.2015.00067 25883585 PMC4383043

[55] Prosperini L Piattella MC Giannì C Pantano P. Functional and structural brain plasticity enhanced by motor and cognitive rehabilitation in multiple sclerosis. Neural Plast. (2015) 2015:481574. 10.1155/2015/481574 26064692 PMC4438192

[56] Tavazzi E Bergsland N Cattaneo D Gervasoni E Lagan,à MM Dipasquale O. Effects of motor rehabilitation on mobility and brain plasticity in multiple sclerosis: a structural and functional MRI study. J Neurol. (2018) 265:1393–401. 10.1007/s00415-018-8859-y 29627940

[57] Bala A Lojek E Marchel A. Cognitive functioning of patients with a PRL-secreting pituitary adenoma: a preliminary report. Neurology. (2016) 86:731–4. 10.1212/WNL.0000000000002252 26701376

[58] Tooze A Hiles CL Sheehan JP. Neurocognitive changes in pituitary adenoma patients after gamma knife radiosurgery: a preliminary study. World Neurosurg. (2012) 78:122–8. 10.1016/j.wneu.2011.09.010 22120272

[59] Lennartsson AK Jonsdottir IH. Prolactin in response to acute psychosocial stress in healthy men and women. Psychoneuroendocrinology. (2011) 36:1530–9. 10.1016/j.psyneuen.2011.04.007 21621331

[60] Vergara-Castaneda E Grattan DR Pasantes-Morales H Perez-Dominguez M Cabrera-Reyes EA Morales T. Prolactin mediates neuroprotection against excitotoxicity in primary cell cultures of hippocampal neurons via its receptor. Brain Res. (2016) 1636:193–9. 10.1016/j.brainres.2016.02.011 26874070

[61] Henry JF Sherwin BB. Hormones and cognitive functioning during late pregnancy and postpartum: a longitudinal study. Behav Neurosci. (2012) 126:73–85. 10.1037/a0025540 21928875 PMC4839972

[62] Torner L Tinajero E Lajud N Quintanar-Stéphano A Olvera-Cortés E. Hyperprolactinemia impairs object recognition without altering spatial learning in male rats. Behav Brain Res. (2013) 252:32–9. 10.1016/j.bbr.2013.05.031 23711928

[63] Montalvo I Gutierrez-Zotes A Creus M Monseny R Ortega L Franch J. Increased prolactin levels are associated with impaired processing speed in subjects with early psychosis. PLoS ONE. (2014) 9:e89428. 10.1371/journal.pone.0089428 24586772 PMC3933530

[64] Gitelman DR Nobre AC Parrish TB LaBar KS Kim YH Meyer JR. A large-scale distributed network for covert spatial attention: further anatomical delineation based on stringent behavioural and cognitive controls. Brain. (1999) 122 (Pt 6):1093–106. 10.1093/brain/122.6.1093 10356062

[65] Morris JS Friston KJ Dolan RJ. Neural responses to salient visual stimuli. Proc Biol Sci. (1997) 264:769–75. 10.1098/rspb.1997.0109 9178546 PMC1688407

[66] Ben-Jonathan N Hnasko R. Dopamine as a prolactin (PRL) inhibitor. Endocr Rev. (2001) 22:724–63. 10.1210/edrv.22.6.0451 11739329

[67] Correale J Farez MF Ysrraelit MC. Role of prolactin in B cell regulation in multiple sclerosis. J Neuroimmunol. (2014) 269:76–86. 10.1016/j.jneuroim.2014.02.007 24612525

[68] Baumann N Pham-Dinh D. Biology of oligodendrocyte and myelin in the mammalian central nervous system. Physiol Rev. (2001) 81:871–927. 10.1152/physrev.2001.81.2.871 11274346

[69] Frago LM Pañeda C Dickson SL Hewson AK Argente J Chowen JA. Growth hormone (GH) and GH-releasing peptide-6 increase brain insulin-like growth factor-I expression and activate intracellular signaling pathways involved in neuroprotection. Endocrinology. (2002) 143:4113–22. 10.1210/en.2002-220261 12239123

[70] Leon-Carrion J Martin-Rodriguez JF Madrazo-Atutxa A Soto-Moreno A Venegas-Moreno E Torres-Vela E. Evidence of cognitive and neurophysiological impairment in patients with untreated naive acromegaly. J Clin Endocrinol Metab. (2010) 95:4367–79. 10.1210/jc.2010-0394 20554710

[71] Luzzi S Pesallaccia M Fabi K Muti M Viticchi G Provinciali L. Non-verbal memory measured by rey–osterrieth complex figure B: normative data. Neurol Sci. (2011) 32:1081–9. 10.1007/s10072-011-0641-1 21630034

[72] Sievers C Samann PG Dose T Dimopoulou C Spieler D Roemmler J. Macroscopic brain architecture changes and white matter pathology in acromegaly: a clinicoradiological study. Pituitary. (2009) 12:177–85. 10.1007/s11102-008-0143-1 18836838 PMC2712618

[73] Carson MJ Behringer RR Brinster RL Mcmorris FA. Insulin-like growth factor I increases brain growth and central nervous system myelination in tTransgenic mice. Neuron. (1993) 10:729–40. 10.1016/0896-6273(93)90173-O 8386530

[74] D'Ercole AJ Ye P O'Kusky JR. Mutant mouse models of insulin-like growth factor actions in the central nervous system. Neuropeptides. (2002) 36:209–20. 10.1054/npep.2002.0893 12359511

[75] Wang X Tong X Zou Y Tian X Sun Z. The impact on cognitive functions of patients with pituitary adenoma before and after surgery. Neurol Sci. (2017) 38:1–7. 10.1007/s10072-017-2980-z 28478494

[76] Bukowczan J Lois K Mathiopoulou M Grossman AB James RA. Reversal of severe cognitive impairment following medical treatment of cystic invasive giant prolactinoma. Endocrinol Diabetes Metab Case Rep. (2016) 2016:150111.2016. 10.1530/EDM-15-0111 26904198 PMC4762223

[77] Kline AE Massucci JL Ma X Zafonte RD Edward Dixon C. Bromocriptine reduces lipid peroxidation and enhances spatial learning and hippocampal neuron survival in a rodent model of focal brain trauma. J Neuro. (2004) 21:1712–22. 10.1089/neu.2004.21.1712 15684763

[78] Dennis M Spiegler BJ Obonsawin MC Maria BL Cowell C Hoffman HJ. Brain tumors in children and adolescents–III. Effects of radiation and hormone status on intelligence and on working, associative and serial-order memory. Neuropsychologia. (1992) 30:257–75. 10.1016/0028-3932(92)90004-6 1574161

